# Self-Attention Based Time-Rating-Aware Context Recommender System

**DOI:** 10.1155/2022/9288902

**Published:** 2022-09-17

**Authors:** Yongfu Zha, Yongjian Zhang, Zhixin Liu, Yumin Dong

**Affiliations:** College of Computer and Information Science Chongqing Normal University, Chongqing, China

## Abstract

The sequential recommendation can predict the user's next behavior according to the user's historical interaction sequence. To better capture users' preferences, some sequential recommendation models propose time-aware attention networks to capture users' long-term and short-term intentions. However, although these models have achieved good results, they ignore the influence of users on the rating information of items. We believe that in the sequential recommendation, the user's displayed feedback (rating) on an item reflects the user's preference for the item, which directly affects the user's choice of the next item to a certain extent. In different periods of sequential recommendation, the user's rating of the item reflects the change in the user's preference. In this paper, we separately model the time interval of items in the user's interaction sequence and the ratings of the items in the interaction sequence to obtain temporal context and rating context, respectively. Finally, we exploit the self-attention mechanism to capture the impact of temporal context and rating context on users' preferences to predict items that users would click next. Experiments on three public benchmark datasets show that our proposed model (SATRAC) outperforms several state-of-the-art methods. The Hit@10 value of the SATRAC model on the three datasets (Movies-1M, Amazon-Movies, Amazon-CDs) increased by 0.73%, 2.73%, and 1.36%, and the NDCG@10 value increased by 5.90%, 3.47%, and 4.59%, respectively.

## 1. Introduction

With the rapid development of the Internet and big data technology, it is more difficult for people to find the items they need [1]. The emergence of a recommender system can solve this problem. It recommends different items to users according to their browsing history so that people can quickly find what they need. Nowadays, recommender systems have been widely used in e-commerce, social media platforms [2], and online platforms.

Context-based sequential recommendation [3] combines the user's recent behavior with the recent intention to capture the next preference from their recent behavior. Among sequential recommendation techniques, the Markov chain is very special. It assumes that the next behavior is only related to the previous behavior, and can accurately predict and recommend the next item. Translation-based approaches [4] model third-order interactions with a TransRec component. Collaborative Filtering (CF) [5] is the first technical method used in recommender systems, which is often combined with Matrix Decomposition (MD). In the collaborative filtering algorithm, the method based on MD attempts to change the dimension to represent the relationship between users' preferences and items' attributes. Then, the inner product between user embedding and item embedding is estimated. The methods based on MD mainly include nonnegative matrix decomposition [6] and decomposition machine [7]. They learn the similarity matrix between items and then make preference recommendations according to the similarity between users' history and their interactive items. However, the method based on the Markov chain extremely depends on the users' last behavior. When the sequence is very long, a large amount of sequential information will be lost, resulting in inaccurate recommendations. At the same time, CF does not consider the order between sequences, cannot capture users' short-term hobbies and long-term hobbies, and also can not recommend items that users have never browsed.

With the development of deep learning technology, recurrent neural networks, and attention mechanisms are gradually used to capture the interests of users in sequential recommendation [8, 9]. The study of [10] uses a recurrent neural network to process sequential data. Due to the recurrent neural network will have a hidden state of the previous step when processing the current state, it has natural advantages in processing sequential data [11]. Because of the great success of the attention mechanism in image processing and natural language processing [12], proposed to combine attention and collaborative filtering to make the next recommendation. [13] combine attention with context. [14] proposed the application of attention mechanism on Gated Recurrent Unit (GRU) to realize sequential recommendation and achieved good results. It classifies the input data format and applies the attention mechanism to the gates of GRU to make changes to make sequential recommendations. NARM [15] employs the attention mechanism on RNN to capture users' main purposes. STAMP [16] uses a novel attention memory network to efficiently capture both the users' general interests and current interests.

Recently, the transformer model [17] has achieved excellent performance and effect on machine translation [18]. It is different from the recurrent neural network (RNN) and convolutional neural network. It is completely based on the self-attention mechanism. [19] proposed the sequential recommendation of the self-attention mechanism, which considers the relative position of items and achieved good results. However, in the process of sequential recommendation, users' preferences and choices will be changed dynamically with the update of the timestamp. [20] models based on absolute time. [21] proposed the time interval aware self-attention of temporal recommendation, which takes the time interval between any two items into account. [22] considers the self-attention of the relative position of items. DT4SR [23] represents items as distributions, which proposes the mean and covariance embedding to model uncertainty in items. [24]proposed a sequential temporal order recommendation based on a temporal graph collaborative transformer. Inspired by the success of graph neural networks (GNN) in other tasks, [25] proposed a framework based on dynamic graph collaborative filtering to capture the relationship between items and users at the same time. [26] proposes a memory augmented graph neural network (MA-GNN) to capture users' long-term and short-term hobbies. [27–29] applied a graph neural network to a sequential recommendation.

Although these methods have achieved convincing results, we believe that they still have some shortcomings. In different periods of sequential recommendation, the user's rating of the item reflects the change in the user's preference, which may affect the user's choice of the next item. Specifically, in different periods, the user's different ratings for the same item or the same type of items show feedback on the dynamic changes of the user's preferences. For example, the user has given low/high ratings to emotional movies at different periods, which may indicate that the user may not like/like watching emotional movies recently. The current research does not take this into account, so this paper proposes a self-attention based time-rating-aware contextual recommendation model to solve this problem. The model considers the influence of users' rating information on items in different periods on users' preferences. The main contributions of this paper are as follows:

- We use a gated recurrent neural network to model the time interval between items and items in the user interaction sequence and use the user information as the initial hidden information to capture the influence of the temporal information on the user's preferences to obtain the temporal context.
- We fuse user interaction sequence information and rating information of items in the interaction sequence to obtain rating context. Then, this paper utilizes the self-attention mechanism to model the temporal context and the rating context to better capture the user's dynamically changing preferences.
- Finally, we conduct experimental studies on three datasets. Extensive experiments show that it outperforms state-of-the-art models.

## 2. Related Work

In this section, we separately review three tasks related to our work: contextual recommendation, GRU, and self-attention.

### 2.1. Contextual Recommendation

The study of [30] pointed out that users' preferences are affected by different types of contexts, and the reasonable use of contextual information by the recommender system is beneficial to the recommended performance. Therefore, they propose the concept of context-aware recommender systems. The study of [14] proposed an attention-based context-aware recommender system. [31] proposed location-aware contextual attention based on session recommendation, which improves recommended performance by considering the location information and content information of items. Due to the excellent performance of the RNN, it has been widely used for sequential recommendation tasks [32, 33]. The study of [21] proposed time-interval-aware self-attention for temporal sequence recommendation, which obtains contextual information by the processing time and then considers the relative position of items. The study of [34] proposed a context-aware citation recommendation based on a bidirectional long short-term memory network (LSTM) with a deep memory network. However, these methods do not consider the effect of item ratings on users' preferences in different periods. In this paper, by utilizing users' information, temporal information, and rating information, we construct temporal context and rating context. The temporal context represents the dynamic change of user preferences over time. Second, the rating context is obtained by fusing the item information and the item's rating information. Finally, we utilize a self-attention mechanism to capture the effects of time and ratings on user's preferences.

### 2.2. GRU and Self-Attention

The study of [14] proposed an attention-based context-aware sequential recommendation model by redefining the update gate and reset gate of the GRU unit. The study of [35] reconstructed GRU and proposed a neural template explanation model for recommendation generation based on GFRU. MTAM [36] exploits temporal awareness for a sequential recommendation. MTAM utilizes GRU to learn short-term intentions and self-attention to learn long-term intentions to capture users' hobbies. CoSAN [37] utilizes self-attention to learn long-term dependencies between collaborative items and generate collaborative session representations. TiSASRec [21] utilizes self-attention to model the absolute positions of items and the time interval between them. This paper utilizes GRU to capture the effect of the time factor on user preferences. Different from these models, the user's information is used as the initial hidden information of the GRU to obtain the temporal context. The user's rating on the item directly reflects the user's liking for the item, which will affect the user's next choice of item. In different periods, the user's rating of the item reflects the change in the user's liking. We utilize a self-attention mechanism to capture the effect of item ratings on users' preferences over different periods.

## 3. Methodology

In this section, we will define the problem of sequential recommendation, along with a description of the embedding layer, the temporal context module, and the SATRAC model. Before that, we briefly introduced the computational method of GRU and attention.  Table 1 shows the symbols used and their descriptions.

Gated recurrent neural networks [38] have been shown to be effective in the sequential recommendation. The GRU consists of an update gate and a reset gate, which is defined as follows:

(1) $$\begin{array}{c} r_{t}=\sigma \left(W_{\text{ir}}x_{t}+b_{\text{ir}}+W_{\text{hr}}h_{\left(t-1\right)}+b_{\text{hr}}\right),\\ z_{t}=\sigma \left(W_{\text{iz}}x_{t}+b_{\text{iz}}+W_{\text{hz}}h_{\left(t-1\right)}+b_{\text{hz}}\right),\\ n_{t}=\text{tanh}\left(W_{\text{in}}x_{t}+b_{\text{in}}+r_{t}\left(W_{\text{hn}}h_{\left(t-1\right)}+b_{\text{hn}}\right)\right),\\ h_{t}=\left(1-z_{t}\right)n_{t}+z_{t}h_{\left(t-1\right)}, \end{array}$$

where *h*_*t*_ is the hidden state at time *t*, *x*_*t*_ is the input at time *t*, *h*_(*t* − 1)_ is the hidden state at time *t* − 1, *r*_*t*_, *z*_*t*_, *n*_*t*_ are the reset state, update state and in the new state, *W* and *b* are coefficients. *σ* is the *sigmoi*  *d* function *σ*(*x*)=1/1(1+exp^−*x*^), tanh is the bitangent tangent curve tanh(*x*)=(exp^*x*^ − exp^−*x*^/exp^*x*^+  exp^−*x*^).

Different from other modeling using recurrent neural networks, we let the initial hidden layer of GRU input user information. By incorporating item information and time interval information into the network, the effect of time on current user preferences is captured as the time interval changes. The self-attention mechanism uses parallel computing and does not need to rely on the hidden layer computation at the previous moment. It is defined as follows:

(2) $$\begin{array}{c} \text{ Attention }=\text{softmax}\left(\frac{QK^{T}}{\sqrt{d}}\right)V, \end{array}$$

where *Q*, *K* and *V* represent query, keyword and value, respectively. In SATRAC model, *Q* stands for rating context, *K* stands for temporal context, and *V* stands for item information.

### 3.1. Problem Formulation

Let *U*, *I*, *S*, and *T* denote the set of user, item, rating, and time, respectively. The purpose of the sequential recommendation is to predict the item *I*_*k*_^*u*^ that the user clicks at the next time *k* when the user's historical item sequence (*I*_1_^*u*^, *I*_2_^*u*^,…, *I*_*k*−1_^*u*^) is known. In the SATRAC model, for each user *u* ∈ *U*, The historical interaction sequence of user *u* is *I*^*u*^ = (*I*_1_^*u*^, *I*_2_^*u*^,…, *I*_∣*I*^*u*^∣_^*u*^), the corresponding temporal sequence and rating sequence are *T*^*u*^ = (*T*_1_^*u*^, *T*_2_^*u*^,…, *T*_|*T*^*u*^|_^*u*^) and *S*^*u*^ = (*S*_1_^*u*^, *S*_2_^*u*^,…, *S*_|*S*^*u*^|_^*u*^), respectively. Generally speaking, the sequential recommendation will limit the length of the sequence, and we will explain how to deal with the length of the sequence later. In the training process, at time *t*, our input is *I*^*u*^ = (*I*_1_^*u*^, *I*_2_^*u*^,…, *I*_*|I*^*u*^*|*−1_), *T*^*u*^ = (*T*_1_^*u*^, *T*_2_^*u*^,…, *T*_*|T*^*u*^*|*−1_^*u*^), *S*^*u*^ = (*S*_1_^*u*^, *S*_2_^*u*^,…, *S*_*|S*^*u*^*|*−1_^*u*^). At time *t* + 1, the next item output we expect is *I*^*u*^ = (*I*_2_^*u*^, *I*_3_^*u*^,…, *I*_|*I*^*u*^|_^*u*^).

### 3.2. Embedding Layer

Before the input of the model, we must convert our data into an embedding representation. For user *u*, use *T*^*u*^=(*T*_1_^*u*^, *T*_2_^*u*^,…, *T*_|*T*^*u*^|_^*u*^) to represent the temporal sequence corresponding to the items in the user interaction sequence, and use *I*^*u*^=(*I*_1_^*u*^, *I*_2_^*u*^,…, *I*_|*I*^*u*^|_^*u*^) represents the user's item sequence, *S*^*u*^=(*S*_1_^*u*^, *S*_2_^*u*^,…, *S*_|*S*^*u*^|_^*u*^) represents the rating sequence corresponding to the item in the user interaction sequence. In the process of embedding representation, we fix the length of our input to *m*. If the length of the user's behavior sequence is less than *m*, then we will add a padding item 0 on the left side. If the length of the user's behavior sequence is greater than *m*, then we Will select the most recent *m* historical behaviors as our input. The embedding layer is as follows:

(3) $$\begin{array}{c} E_{T}^{u}=\text{ Embedding }\left(T^{u}\right),\\ E_{I}^{u}=\text{ Embedding }\left(I^{u}\right),\\ E_{u}^{u}=\text{ Embedding }\left(u\right),\\ E_{S}^{u}=\text{ Embedding }\left(S^{u}\right), \end{array}$$

where *E*_*T*_^*u*^ ∈ *R*^*m*×*d*^ represents the embedding of the corresponding temporal sequence in the user *u* interaction sequence, *E*_*I*_^*u*^ ∈ *R*^*m*×*d*^ represents the embedding of the item corresponding to user *u*, *E*_*u*_^*u*^ ∈ *R*^1×*d*^ represents the embedding of user *u*, *E*_*S*_^*u*^ ∈ *R*^*m*×*d*^ represents the embedding of the corresponding rating sequence in the user *u* interaction sequence.

### 3.3. Temporal Context Module

**Figure 1:**
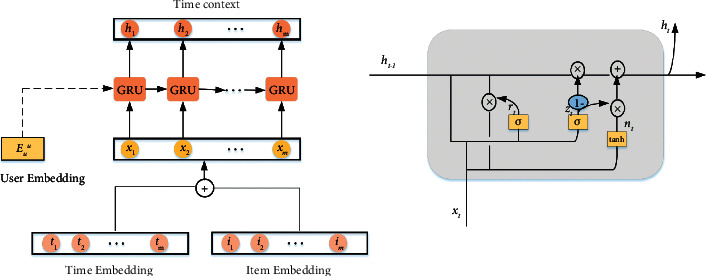
The figure on the left shows the gated neural network used to obtain the temporal context, and the figure on the right is the internal structure of the GRU.

The method adopted by [19] discards the timestamp, assuming that the time interval between all items is the same, and only retains the user's behavior sequence. Recently, [21] achieved good results by modeling the absolute positions of items and their time intervals in the sequence. [14] proposed to use the attention mechanism on the GRU and make improvements to use time in the recommendation. We build a network through the Gated Recurrent Unit (GRU) to capture the influence of the temporal sequence on the user's behavior. In the processing of time, the difference between the time of two adjacent items is used to represent the time information. We also input the user's information into the GRU network, because we think that for different users, even if the behavior sequence and time series are the same, the final recommendation should be different. The temporal context module is shown in Figure 1.

The following are the initial values of the input and hidden layers:

(4) $$\begin{array}{c} h_{0}=E_{u}^{u},\\ x^{t}=e_{t}^{T}+e_{t}^{I}, \end{array}$$

where *e*_*t*_^*T*^ represents the time embedding of the user at time *t*, and *e*_*t*_^*I*^ represents the item embedding at time *t*. We can get a time context *C*_*T*_^*u*^ through the network:

(5) $$\begin{array}{c} C_{T}^{u}=\left(h_{1},h_{2},\ldots ,h_{m}\right), \end{array}$$

where *C*_*T*_^*u*^ is the temporal context that contains user information and item time information.

### 3.4. SATRAC Modle

**Figure 2:**
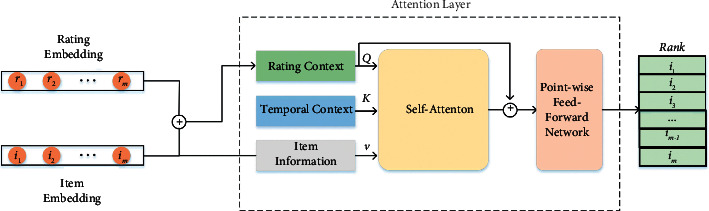
The architecture of our proposed SATRAC.

The user's rating of an item can be seen as displayed feedback. The rating can reflect the user's hobby for the current item, which greatly affects the user's next choice of item. First, we fuse the rating information of the item with the user interaction sequence information to obtain the rating context. In different periods, to better capture the impact of rating on users. We utilize a self-attention mechanism to capture the effects of temporal context and rating context on user hobby.  Figure 2 shows the structure of the SATRAC model.

The SATRAC model includes self-attention layers, residual connections, layer normalization, and pointwise feedforward neural networks.

(6) $$\begin{array}{c} C_{S}^{u}=E_{S}^{u}+E_{I}^{u}, \end{array}$$

where *C*_*S*_^*u*^ ∈ *R*^*m*×*d*^ represents the rating context that fuses the item information in the interaction sequence and the corresponding rating information of the item in the interaction sequence.

(7) $$\begin{array}{c} Z^{u}=\text{Attention}\left(C_{S}^{u},C_{T}^{u},E_{I}^{u}\right), \end{array}$$

where *Z*^*u*^ ∈ *R*^*m*×*d*^, *Q* represents the rating context, *K* represents the temporal context, and *V* represents the item information.

#### 3.4.1. Residual Connection

In the past, to better extract features, more often they used the superposition of multilayer neural networks [39]. However, in some cases, multilayer neural networks did not have better performance until the emergence of residual networks [40]. The main idea of the residual network is to transfer low-dimensional features to high-dimensional through residual connection. In this paper, the rating context information and the information captured by the self-attention mechanism are residually connected.

(8) $$\begin{array}{c} Z^{u}=Z^{u}+C_{S}^{u}. \end{array}$$

#### 3.4.2. Layer Normalization

To enhance the capabilities of the model and accelerate neural network training [41], the statistical data used in layer standardization is independent of other samples in the same batch. The operation is defined as follows:

(9) $$\begin{array}{c} Z^{u}=\text{LayerNorm}\left(Z^{u}\right), \end{array}$$

where LayerNorm (*x*) is defined as follows:

(10) $$\begin{array}{c} \text{LayerNorm}\left(x\right)=\alpha ⊙\frac{x-\mu }{\sqrt{\sigma^{2}+\epsilon }}+\beta , \end{array}$$

where ⊙ is the elementwise product, *μ* and *σ* are the mean and variance of *x*, and *α* and *β* are the scale factors and bias terms, respectively.

#### 3.4.3. Pointwise Feed-Forward Network

Although self-attention can aggregate temporal and rating contexts with adaptive weights, it is ultimately a linear model. To give the model nonlinearity and consider the interaction between different potential dimensions, we adopted the GELU function [42]. To solve the model's overfitting, we used the dropout technique [43].

(11) $$\begin{array}{c} \text{FFN}\left(Z^{u}\right)=\text{Dropout}\left(\text{GELU}\left(Z^{u}\right)\right). \end{array}$$

Finally, after stacking self-attention layers, we obtain the following equation:

(12) $$\begin{array}{c} Z^{u}=\left(z_{1}^{u},z_{2}^{u},\ldots ,z_{m}^{u}\right), \end{array}$$

where *Z*^*u*^ represents the embedded information of user *u* representing the click on the next item.

### 3.5. Prediction Layer

Like the [19, 21] model, to predict the next item the user clicks, we rank the items using the following computation:

(13) $$\begin{array}{c} {\text{Rank}}_{i}^{u}=Z^{u}M_{i}^{I}, \end{array}$$

where *M*_*i*_^*I*^ ∈ *R*^*d*^ is the embedding of item *i*. Finally, get the ranking of *m* items, and then recommend the top-ranked items to user *u*.

### 3.6. Model Training and Loss Function

The sequence of items input for our model training is: *I*^*u*^=(*I*_1_^*u*^, *I*_2_^*u*^,…, *I*_*|I*^*u*^*|*−1_^*u*^), and then a fixed length *I*^*u*^=(*I*_1_^*u*^, *I*_2_^*u*^,…, *I*_*m*_^*u*^), we expect the output at time *t* to be:

(14) $$\begin{array}{c} O_{i}=\left\{\begin{array}{cc} \langle \text{pad}\rangle & \text{ if }I_{i}\text{ is a padding item }\\ I_{t+1} & 1\leq t<m\\ I_{|I^{u}|}^{u} & t=m \end{array}\right., \end{array}$$

where <pad> represents the padding item. *O*_*t*_ represents the output we expect. We use binary cross entropy loss as the objective function:

(15) $$\begin{array}{c} -\sum_{{ℐ}^{u}\in ℐ}\sum_{t\in \left[1,2,\ldots ,m\right]}\left[\log\left(\sigma \left(R_{O_{t},t}\right)\right)+\sum_{j\notin {ℐ}^{u}}\log\left(1-\sigma \left(R_{j,t}\right)\right)\right]. \end{array}$$

For padded items, ignore its loss. Our model uses Adam optimizer [44].

## 4. Experiment

In this section, we validate the effectiveness of the SATRAC model on three datasets. First, we introduce our experimental setup and compare it with current state-of-the-art baselines. Then, we discuss the effect of some hyperparameters on the SATRAC model. Finally, we discuss the impact of temporal information and rating information on the SATRAC model.

### 4.1. Dataset

We evaluate our method on three real datasets. These datasets have different sizes, and different sparsity, and are all common datasets. As shown in Table 2.

Movies-1M https://www.kaggle.com/datasets/odedgolden/movielens-1m-dataset: it contains 1 million movie records rated by users.

Amazon https://www.kaggle.com/datasets/deovcs/amazon-dataset: we use the Amazon-Movies and Amazon-CDs datasets from the series of datasets presented in [45].

All three datasets include user ratings and timestamps, and we filter out cold-start users and users with less than 3 interaction sequences. When calculating the time interval between adjacent items, we set a maximum time interval value. If the time interval of adjacent items in the user interaction sequence is greater than the time interval value we set, then we set the time interval of adjacent items to a fixed value. On the three datasets, the maximum interval value we set is 2048. As proposed in [19], for each user, we use the latest item in the interaction record for testing, the second-to-last item for validation, and the remaining items for training.

### 4.2. Evaluation Index

We use two frequently used metrics Hit@10 and NDCG@10 to evaluate the performance of the model [46]. Hit@10 calculates the ratio of basic fact items in the top 10 items. NDCG@10 will consider location and assign higher weights to higher locations. For each user *u*, we randomly sample *m* − 1 negative items and rank these items with basic fact items. We calculate Hit@10 and NDCG@10 based on the ranking of these *m* items (This is slightly different from [19, 21]).

### 4.3. Baselines

To demonstrate the effectiveness of the SATRAC model, we compare SATRAC with the following baselines, which include some classic baselines and some state-of-the-art baselines.

- **BPR** [47]: the bayesian ranking is personalized and often used as a matrix factorization recommender.
- **GRURec** [11]: it uses RNN to model user action sequences for session-based recommendations. Regard each user's feedback sequence as a conversation, and then perform a recommended algorithm.
- **Caser** [48]: a convolution-based algorithm designed to capture the general preferences and order patterns of users.
- **SASRec** [19]: a sequential recommendation model based on a self-attention mechanism.
- **MARank** [49]: this method considers recent items and applies multi-order attention to capture single and joint-level item relevance.
- **HGN** [50]: a sequential model that includes feature selection and instance selection modules combined with Bayesian personalized ranking (BPR) to obtain long-term and short-term user interests.
- **TiSASRec** [21]: a recently proposed state-of-the-art model, this model mainly considers self-attention sequential recommendation for time interval modeling.
- **SATRAC-NR**: a variant of the SATRAC model. SATRAC-NR does not add temporal context information and replaces temporal context information with rating context information for calculation. This is beneficial to show the impact of temporal context information on the SATRAC model.
- **SATRAC-NS**: a variant of the SATRAC model. SATRAC-NS does not add rating information and replaces rating context information with time context information for calculation. This is beneficial to show the impact of rating context information on the SATRAC model.

**Figure 3:**
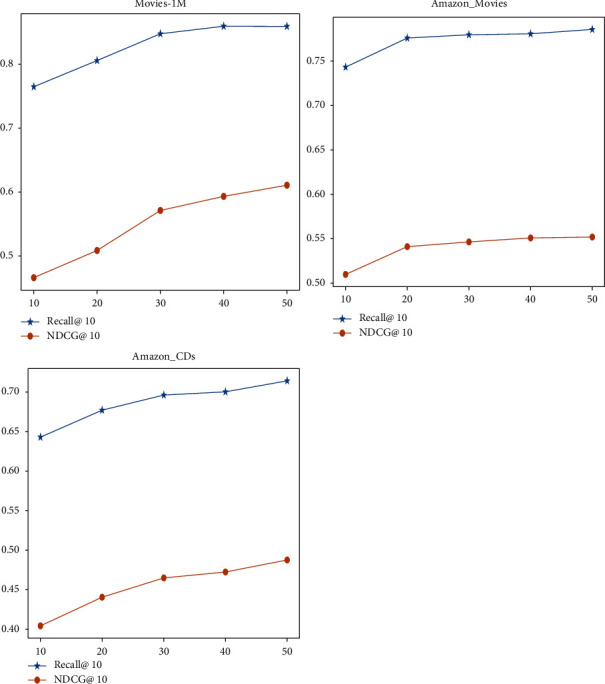
The influence of different dimensions *d*.

**Figure 4:**
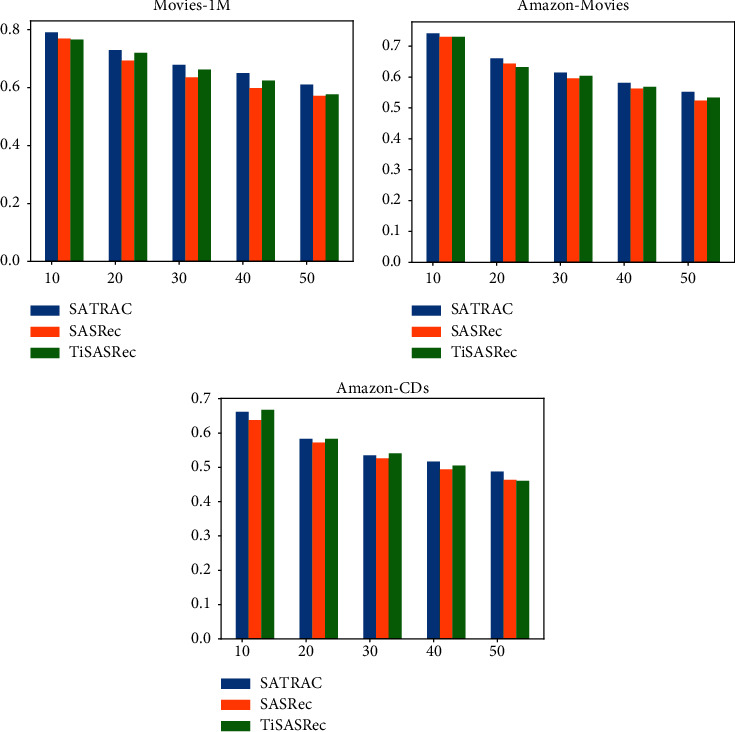
The effect of different sequential length *m* on recommended performance (NDCG@10).

For some comparative baselines, we used the same dataset and we directly report the experimental results of their paper. For other baselines using the self-attention mechanism, we reproduce the model under the premise that the model parameters are set optimally to obtain the best performance of the model. For the fairness of the comparison, our model uses the common framework pytorch and the same optimization algorithm adam. All datasets used a learning rate of 0.001 and were trained the same number of times. The number of self-attention heads and self-attention layers is both set to 1, the value of dropout is 0.2, and we set both *m* and *d* to 50. To discuss the impact of embedding dimension *d* and sequence length *m* on the model recommended performance, we do some comparative experiments. The *m* value is selected as [10, 20, 30, 40, 50], and the potential dimension *d* is selected as [10, 20, 30, 40, 50], and different values are compared. If all metrics are lower than the last evaluation metrics, the training is terminated. The maximum number of training sessions is 200. During implementation, the batch size used is 128, and all experiments are performed with a GTX-1650 Ti graphics processor. This paper discusses the impact of embedding dimension *d* and sequential length *m* on the model recommended performance in Figures 3 and 4, respectively.

### 4.4. Recommended Performance

Table 3 shows the recommended performance of all baselines on the three datasets. On Movies-1M, Amazon-Movies, and Amazon-CDs datasets, the SATRAC model shows the state-of-the-art recommended performance. Among the baseline methods, MARank has state-of-the-art performance compared with other baselines. MARank can capture individual-level, and union-level user interactions and tackle sparse datasets well. On the dense dataset Movies-1M, the recommended performance of the self-attention-based baseline is significantly better than the ordinary baseline. Because the self-attention mechanism has a stronger ability to capture long-term sequential models. On Movies-1M and Amazon-Movies datasets, the TiSASRec model outperforms SASRec, which shows that utilizing temporal information can help improve recommended performance. HGN outperforms TiSASRec on the Amazon-CDs dataset, which may be because the HGN model can capture both the long-term and short-term hobbies of users. On the three datasets, SATRAC outperforms the best baseline methods on both metrics. On the one hand, SATRAC takes into account the influence of temporal information on user preferences, utilizing a gated neural network to obtain temporal context. On the other hand, we consider the impact of user ratings on items on users' preferences. Integrate item information and rating information to obtain context. Finally, SATRAC utilizes a self-attention mechanism to capture the effects of temporal context and rating context on users' preferences. Most of the existing baselines only consider the impact of time information on users' preferences.

### 4.5. Influence of Hyperparameters

In Figure 3, we discuss the effect of the embedding dimension *d* on Hit@10 and NDCG@10, while setting other hyperparameters to be optimal. The value of the embedding dimension *d* is [10, 20, 30, 40, 50]. On the three datasets, the recommended performance of the SATRAC model will gradually improve. This is because as the dimension *d* becomes larger and larger, more feature information can be represented, but when the *d* value reaches a certain value, the *d* value has a limited impact on the model, and the final model will tend to be stable, eventually, the model will stabilize. In Figure 4, we discuss the effect of sequential length *m* on NDCG@10, while setting other hyperparameters to be optimal. The value of the sequential length *m* is [10, 20, 30, 40, 50]. When the value of *m* becomes larger, the recommended performance of the SATRAC model, SASRec model, and TiSASRec model starts to decrease. This is because a lot of noise is generated as the sequential length increases, which can lead to lower recommendation model performance. Compared with the SASRec model and the TiSASRec model, the recommended performance of the SATRAC model degrades more slowly, which shows the importance of rating information and temporal information in the sequential recommendation, and also shows the effectiveness of the SATRAC model in capturing time information and rating.

### 4.6. Discussion

The effects of time information and rating information on the recommended performance of the SATRAC model are shown in Table 4. On the three datasets, the SATRAC-NT model outperforms the SASRec model. This illustrates that rating information is important for sequential recommendation tasks, and the SATRAC-NT model is effective in capturing rating information. On the three datasets, the SATRAC-NR model outperforms the TiSASRec model, which demonstrates the effectiveness of the SATRAC-NR model in capturing temporal information. Compared with the TiSASRec model, the SATRAC-NR model first uses the GRU network to capture the user's temporal interaction information and the user's information and then uses the self-attention mechanism to further capture the impact of temporal information on the user's preferences. The SATRAC model outperforms the SATRAC-NT model and the SATRAC-NR model, which indicates that the SATRAC model is reasonable and effective to capture the user's time and rating information.

## 5. Conclusion

This paper proposes a self-attention-based time-rating-aware context recommender system model (SATRAC). The SATRAC model first uses a gated recurrent neural network to model the user interaction sequence and the time interval information of items in the interaction sequence to capture the impact of time information on user preferences, thereby obtaining the time context. Then, the user interaction sequence information and the rating information of the items in the sequence are fused. Finally, a self-attention mechanism is used to learn the effects of temporal context and rating context on user preferences. Extensive experiments on the three datasets show that the SATRAC model outperforms state-of-the-art models, which demonstrates the superiority of the SATRAC model in processing data with time information and rating information. Current research and future research must be discussed under the premise of ensuring user privacy and security. In future research, we will also explore the influence of some other item attributes (such as item description and item image) on sequence recommendation, so that the sequence recommendation model has a more powerful recommendation performance.

## Figures and Tables

**Table 1:** Notation.

Notation	Description
*U*, *I*, *R*, *T*	Set of users, items, ratings and time
*I* ^*u*^	Item sequence for user *u*
*T* ^*u*^	Time sequence of items for user *u*
*R* ^*u*^	Rating sequence of items for user *u*
*m*	The maximum length of the sequence
*d*	Embedding dimension
*C*_*T*_^*u*^	Time context information of user *u*
*C*_*R*_^*u*^	Rating context information of user *u*
*O*_*t*_	Output of the model at time *t*

**Table 2:** Dataset statistics.

Dataset	Users	Items	Interactions
Movies-1M	6040	3416	999611
Amazon-Movies	40928	37654	1163413
Amazon-CDs	26875	42779	770188

**Table 3:** Comparison of recommended performance of the SATRAC model and other baselines. The best results are in bold. The underlined numbers are suboptimal results.

Dataset	Metric	BPR	GRURec	Caser	MARank	SASRec	HGN	TiSASRec	SATRAC
Movies-1M	Hit@10	0.5922	0.5781	0.7487	0.7622	0.8493	0.8123	0.8529	**0.8591**
	NDCG@10	0.3401	0.3781	0.5123	0.5169	0.5712	0.5448	0.5767	**0.6107**

Amazon-movies	Hit@10	0.5517	0.5368	0.4938	0.6546	0.7563	0.7429	0.7647	**0.7856**
	NDCG@10	0.3464	0.3317	0.3201	0.4562	0.5238	0.5167	0.5334	**0.5519**

Amazon-CDs	Hit@10	0.5627	0.5364	0.6865	0.6981	0.7015	0.7045	0.7001	**0.7141**
	NDCG@10	0.3626	0.3267	0.4585	0.4589	0.4635	0.4662	0.4604	**0.4876**

**Table 4:** We discuss the impact of temporal information and rating information on SATRAC models.

Dataset	Metric	SATRAC-NT	SATRAC-NR	SATRAC
Movies-1M	Hit@10	0.8529	0.8561	**0.8591**
	NDCG@10	0.5812	0.0.6012	**0.6107**

Amazon-movies	Hit@10	0.7782	0.7717	**0.7856**
	NDCG@10	0.5421	0.5406	**0.5519**

Amazon-CDs	Hit@10	0.7066	0.7005	**0.7141**
	NDCG@10	0.4742	0.4714	**0.4876**

## Data Availability

The data used to support the study are included in the paper.https://www.kaggle.com/datasets/odedgolden/movielens-1m-dataset. https://www.kaggle.com/datasets/deovcs/amazon-dataset.
